# Pressurized Hot Water Extraction and Bio-Hydrogels Formulation with *Aristotelia chilensis* [Mol.] Stuntz Leaves

**DOI:** 10.3390/molecules26216402

**Published:** 2021-10-23

**Authors:** Audrey Bianchi, Pamela R. Rivera-Tovar, Vanesa Sanz, Tania Ferreira-Anta, María Dolores Torres, José Ricardo Pérez-Correa, Herminia Domínguez

**Affiliations:** 1 Department of Chemical Engineering, Faculty of Sciences, University of Vigo, Edificio Politécnico, As Lagoas s/n, 32004 Ourense, Spain; audrey.tiare@hotmail.fr (A.B.); vsanz@uvigo.es (V.S.); ta.ferreiraan@gmail.com (T.F.-A.); 2 EPF–School of Engineering, 21 Boulevard Berthelot, 34000 Montpellier, France; 3 Chemical and Bioprocess Engineering Department, School of Engineering, Pontificia Universidad Católica de Chile, Santiago 7820436, Chile; pqrivera@uc.cl (P.R.R.-T.); perez@ing.puc.cl (J.R.P.-C.)

**Keywords:** maqui, phenolic compounds, microwave, heating methods, gelling agent, rheology

## Abstract

*Aristotelia chilensis* is a plant rich in phenolics and other bioactive compounds. Their leaves are discarded as waste in the maqui berry industry. A new application of these wastes is intended by the recovery of bioactive compounds using pressurized hot water extraction with conventional or microwave heating. Both technologies have been selected for their green character regarding the type of solvent and the high efficiency in shorter operation times. Extractions were performed in the temperature range 140–200 °C with a solid/liquid ratio of 1:15 (*w:w*). The extracts’ total phenolic content, antioxidant capacity, and saccharides content obtained with both heating methods were measured. Additionally, the thermo-rheological properties of the gelling matrix enriched with these extracts were analyzed. Optimum conditions for lyophilized extracts were found with conventional heating, at 140 °C and 20 min extraction; 250.0 mg GAE/g dry extract and 1321.5 mg Trolox/g dry extract. Close to optimum performance was achieved with microwave heating in a fraction of the time (5 min) at 160 °C (extraction), yielding extracts with 231.9 mg GAE/g dry extract of total phenolics and antiradical capacity equivalent to 1176.3 mg Trolox/g dry extract. Slightly higher antioxidant values were identified for spray-dried extracts (between 5% for phenolic content and 2.5% for antioxidant capacity). The extracts obtained with both heating methods at 200 °C contained more than 20% oligosaccharides, primarily glucose. All the formulated gelling matrices enriched with the obtained extracts displayed intermediate gel strength properties. The tested technologies efficiently recovered highly active antioxidant extracts, rich in polyphenolics, and valuable for formulating gelling matrices with potential applicability in foods and other products.

## 1. Introduction

*Aristotelia chilensis*, commonly known in South America as maqui, is a native tree of the subantarctic forests of Chile and Argentina, growing at 2500 m above sea level and reaching approximately 4–5 m height [1]. It produces succulent berries, which have been applied throughout civilizations to prepare medicines, foods, and colorants. Infusions of maqui leaves that are highly antioxidant and rich in phenolic compounds [2] were used by ancient cultures to treat several ailments, such as diarrhea, throat infections, and mouth ulcers [3]. Nowadays, these infusions are still used as a natural muscle relaxant or as an analgesic [1]. Insunza et al. [4] has shown that maqui leaves extracts are potent pesticides as well as anti-inflammatory and cardio-protective agents. Despite all these well-documented properties, maqui leaves are discarded as waste in the maqui berry industry. 

Traditional and modern technologies have been used to add value to agro-industrial discards, fostering a circular economy and mitigating their negative environmental impact. Microwave-assisted extraction (MAE) and pressurized hot water extraction (PHWE) are relatively new technologies that have been applied to leach out bioactive compounds and phytonutrients [5] from several matrices, requiring less energy and solvent, and in shorter times than conventional methods [6]. Both offer significant advantages as greener extraction techniques, including the use of water as extracting solvent and the possibility of attaining higher extraction yields and higher product selectivity, as well as the reduction in operation times, which in the case of microwave extraction also represents a reduction in energy consumption. 

Some recent studies have focused on developing natural polymeric hydrogels enriched with bioactive compounds with potential applications in the food and non-food fields. These hydrogels are preferred to other matrices since they are soft, highly absorbent, flexible, and moldable [7]. Many daily life goods, such as foods, pharmaceuticals, cosmetics, and agricultural products, contain bioactive hydrogels given their low cost, harmlessness, and environmental friendliness [8]. The development of alternative gelling matrices incorporated with soluble extracts with potential bioactive features from alternative residual sources allows not only the revalorization of the raw materials following a biorefinery and circular economy approach [5,6] but also the alleviation of the current market demand for natural antioxidant extracts [1,5,7,8,9]. Thus, novel formulations could be attractive bases for gelling desserts, fortified functional food, or edible films within the food sector, as well as for personal care products with potential health benefits [8,9].

This study aimed to design a valuable product from maqui leaf discards. PHWE, with two heating methods (conventional and microwave), were optimized to obtain liquid polyphenolic extracts. As far as we know, this is the first time that the impact of PHWE temperature on the carbohydrate content of *A. chilensis* extracts has been assessed. These extracts were used to formulate bioactive hydrogels, and their rheological properties were analyzed. 

## 2. Results and Discussion

**Figure 1:**
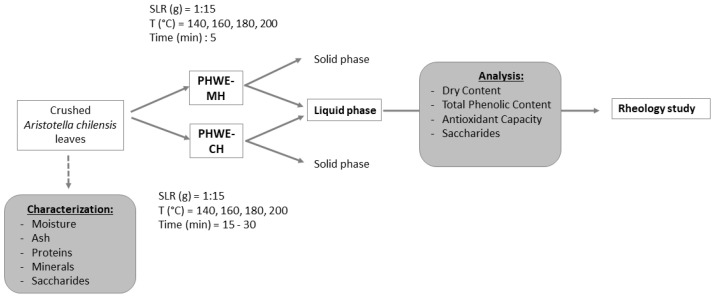
Processing conditions of pressurized hot water extraction with microwave heating (PHWE-MH) and conventional heating (PHWE-CH) of bioactive compounds from *Aristotelia chilensis* leaves. SLR: solid–liquid ratio (*w/w*).

Pressurized hot water extractions with microwave heating (PHWE-MH) and conventional heating (PHWE-CH) were used to recover bioactive compounds from *A. chilensis* discarded leaves in a range of commonly used conditions for similar raw materials [9]. A schematic of the process and the subsequent analysis are summarized in Figure 1.

### 2.1. Characterization of the Raw Material

Table 1 shows the fundamental characterization of the tested maqui leaves. Previous studies by Rivera-Tovar et al. [10] showed that the raw material contained around 10% moisture, low amounts of ash (6%), and a considerable amount of protein (15%). The chemical analysis of minerals considered essential for cellular wellness revealed a strong presence of calcium (21.23 g/kg), followed by potassium (10.50 g/kg) and magnesium (1.98 g/kg). Other minerals such as iron (237.9 mg/kg), sodium (20.14 mg/kg), zinc (12.33 mg/kg), copper (<7 mg/kg), cadmium (<5 mg/kg), and lead (< 10 mg/kg) were present at moderate levels, acceptable for human consumption. Damascos and Arribere [11] found that young leaves of *A. chilensis* are potentially rich in salts such as Mg, K, Mn, Na, and Ca, suggesting that infusions of this plant should not be consumed frequently. The total carbohydrate content represented 37.6% of the dry mass, where 15.6% was mainly glucose, followed by a combination of xylose + galactose + mannose (10.3%), rhamnose (2%), and arabinose (2%), as well as a small proportion of fucose, ribose, and formic and acetic acid, which added up to about 6% of total carbohydrates. Other reports showed that alkaloids are the main bioactive compounds of this traditional herbal medicine [12,13,14], which also contains ursolic acid [15], an antidepressant, collagen skin production stimulator, and nervous system protector [16].

### 2.2. Extraction

**Figure 2:**
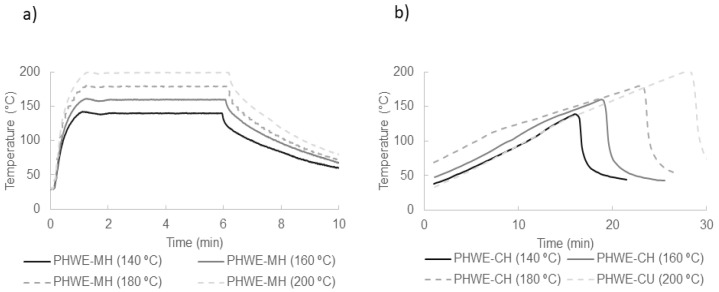
Temperature evolution in PHW extraction of *A. chilensis* leaves using (**a**) microwave and (**b**) conventional heating, operating at 140, 160, 180, and 200 °C.

Figure 2 shows the heating and cooling profiles recorded during PHWE operation using microwave and conventional heating in the temperature range 140–200 °C. A different temperature evolution is evident. While the temperature rise takes about one minute in the PHWE-MH process, the temperature rise in PHWE-CH is linear and slow, taking between 15 and 30 min to reach the set temperature. To compare both heating methods better, we computed the severity, a combined factor of time and temperature. In Table 2, it can be seen that the severity values for PHWE-MH were in the range 1.36–2.56, while for PHWE-CH were in the range 1.52–3.48. 

### 2.3. Liquid Phase Composition

#### 2.3.1. Total Extraction Yield and Phenolic Content

**Figure 3:**
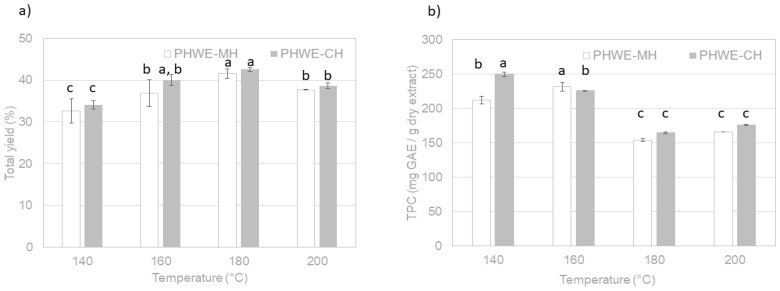
Influence of temperature on (**a**) soluble solids extraction yield (%) and (**b**) total phenolic content (mg GAE/g dry extract) during PHWE-MH and PHWE-CH of *A. chilensis* leaves. Letters above the graph bars indicate significantly different values (95% confidence, *p* < 0.05).

The impact of temperature on the extraction yield (g dry extract/g of dry leaves) and the total phenolic content (mg GAE/g dry extract) using both heating methods are shown in Figure 3. Extraction yields did not present significant differences with the tested heating methods, and the optimum temperature was 180 °C. Probably, both heating methods generated extracts with particular phenolic compositions due to the different extraction temperature profiles. PHWE-MH could favor the recovery of phenolic compounds such as chlorogenic and coumaric acids, which are present in maqui leaves and require high temperatures for their release [15]. In contrast, PHWE-CH could prevent the degradation of heat-sensitive phenolic compounds such as gallic acid and catechin [15] since this extraction process is carried out at lower average temperatures. Comparable results have been found for fruits in [17], where the optimum temperature in microwave-assisted extraction of lyophilized maqui berries was 100 °C (maximum tested value), achieving 54.27 mg/g of phenolic compounds, regardless of the extraction time, using a water/methanol mixture as a solvent. Similarly, Rivera-Tovar et al. [10] showed that more phenolic compounds were recovered with increasing HPLE temperature from 80 to 200 °C, achieving a total of 208.94 mg GAE/g dry leaves (200 °C, 20% ethanol, 5 min, and 5 cycles), whereas maximum purity (400.7 mg GAE/g dry extract) was attained at 80 °C using 80% ethanol in one cycle.

Based on Rivera-Tovar et al. [10], the major phenolic compounds found in maqui leaves were flavonols (41–48%), phenolic acid (35–45%), flavanols (4–22%), and others (1–7%). In our experiments, higher phenolic contents were obtained at the lowest tested temperatures (140–160 °C), which would also be more favorable in terms of energy. It was possible to achieve a maximum of 231.9 mg GAE/g dry extract (approx. 86 mg GAE/g dry leaves) using microwave heating at 160 °C, and 250.0 mg GAE/g dry extract (approx. 85 mg GAE/g dry leaves) using conventional heating at 140 °C. Since the extraction of non-phenolic compounds is favored at higher temperatures (180–200 °C), more soluble solids are recovered (higher yields), but the overall recovery of phenolic compounds is significantly reduced, probably due to hydrolysis. Note here that the above data (Figure 3 b) corresponded with those obtained after lyophilization of the extracted liquid phases as representative of both selected drying methods. It should be indicated that spray-dried extracts exhibited the same phenolic content trends, although with values of this parameter around 5% higher. A more traditional extraction technique, such as ethanol maceration [18], recovered 15 times fewer total phenolic compounds from maqui leaves (15.9 mg GAE/g dry extract) than the green technologies used in our study.

#### 2.3.2. Radical Scavenging Capacity

**Figure 4:**
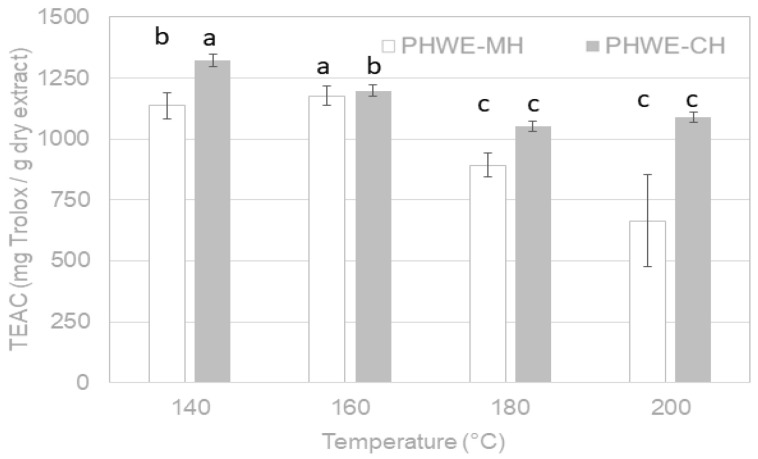
Antioxidant capacity of extracts influenced by temperature during PHWE-MH and PHWE-CH of *A. chilensis* leaves expressed as mg Trolox/g dry extract. Letters above the graph bars (i.e., a,b,c) indicate significantly different values (95% confidence, *p* < 0.05).

The ABTS radical scavenging capacity, expressed as TEAC values in mg Trolox/g dry extract, of the *A. chilensis* leaf extracts obtained by PHWE-MH and PHWE-CH is shown in Figure 4. Again, the impact of extraction temperature on lyophilized extracts is presented in this plot as representative of both tested drying methods used previously to the antioxidant features determination. As expected, a similar tendency as the total phenolic compounds behaviors was identified, with antioxidant capacity values around 2.5% higher for spray-dried extracts. The maximum antioxidant capacity was obtained at the lowest tested temperatures (140–160 °C) with an average of 1176.3 mg Trolox/g dry extract for PHWE-MH (160 °C) and 1321.5 mg Trolox/g dry extract for PHWE-CH (140 °C). Operating with hot pressurized liquid extraction (HPLE) using water/ethanol solutions as a solvent, Rivera-Tovar et al. [10] attained a maximum antioxidant capacity of 1668.02 mg TEAC/g dry extract at 122 °C, 5% ethanol, 5 min, and 3 cycles.

Other authors have confirmed that clean technologies were more efficient and energy-saving than traditional methods. Studies performed with *Elaeocarpus tuberculatus* and *Elaeocarpus ganitrus* leaves (plants of the same family as *A. chilensis*) showed that the antioxidant capacities reached by the Soxhlet apparatus and orbital shaker method were 10 times lower than microwave and high-pressure extraction technologies [19,20].

#### 2.3.3. Saccharides and Derivatives Content

**Figure 5:**
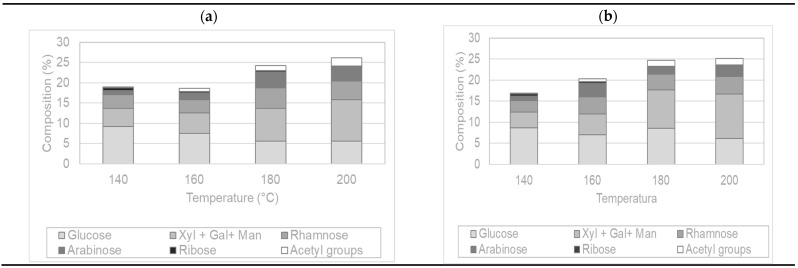
Soluble oligomers and other groups found in the extracts obtained with (**a**) PHWE-MH and (**b**) PHWE-CH at different temperatures.

Figure 5 shows the influence of the extraction temperature on the solubilization of sugar components from maqui leaves. No significant differences between the two heating methods were observed, presenting very similar trends and results. The oligosaccharides’ content increased with increasing temperature, up to 200 °C, reaching maximum values of 28.2% and 26.7% content with PHWE-MH and PHWE-CH, respectively. 

In both cases, glucose solubilization was favored at the lowest tested temperatures, reaching the highest content at 140 °C (around 9%), and then dropped when the temperature rose. This decrease in glucose content could be attributed to hydroxymethylfurfural (HMF) formation from glucose, which usually occurs at temperatures above 130 °C [21,22]. HMF formation could be favored with microwave heating since the extracts’ decrease in glucose content is evident. In turn, less HMF could have been formed with conventional heating since high temperatures (180 and 200 °C) lasted a few seconds in these extractions. The xylose + galactose + mannose group showed an opposite behavior, increasing their content with rising extraction temperatures, reaching about 10% in both cases. The acetic group content was more abundant between 180 and 200 °C, reaching values close to 2%. The results are consistent with previous studies using agro-industrial discards [23], showing the influence of operation temperature on the degradation of both phenolic compounds and some saccharidic constituents found in the oligomeric configuration, particularly those containing glucose.

### 2.4. Rheological Properties of Enriched Hydrogels

**Figure 6:**
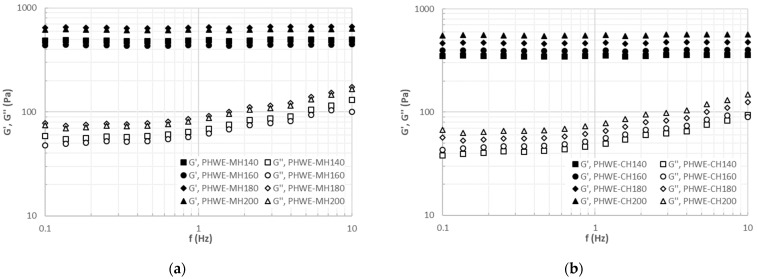
Viscoelastic profiles of gelling matrices prepared with liquid extracts from (**a**) PHWE-MH and (**b**) PHWE-CH and carrageenan (1%, 0.1 mol/L KCl) as a gelling agent.

Figure 6 shows the influence of enriching hybrid carrageenan-based matrices with extracts obtained with both heating methods. All formulated matrices, independent of the extracts added, exhibited gel behavior with elastic modulus, G’, higher than the viscous modulus, G’’, almost invariant over the tested frequency range. This behavior is consistent with previous results with gelling matrices formulated with natural polymers and enriched with antioxidant leaf extracts [9,24]. At fixed frequency, the elastic modulus was about 10 times higher than the viscous one in all cases. Note here that the magnitude of the viscoelastic moduli was slightly higher for matrices enriched with extracts obtained with microwave heating. This behavior agrees with the above soluble extract yields, suggesting a higher competition for available water between the higher carrageenan and antioxidant soluble extracts. It can be observed that the gel strength, in terms of G’ and G’’ moduli, increased with increasing temperature in PHWE-CH extracts.

The highest viscoelastic enhancement was observed between 160 and 180 °C. In the case of matrices made with microwave heating extracts, a similar overall tendency was identified. However, in this particular case, a maximum weakening of the gels was found at 160 °C. Again, this is consistent with the aforementioned antioxidant profiles (Figure 3 and Figure 4), highlighting the relevance of gaining insight into the impact of different extraction methods on the mechanical properties of the developed matrices for the adequate selection of the final application and the corresponding manufacturing stage. All proposed matrices displayed intermediate gel strength features, with potential applications in food [25] and non-food products [25].

## 3. Materials and Methods

### 3.1. Raw Material

Maqui leaves from adult plants (4 years old) were collected from La Araucanía (Chile) in May 2016, subsequently dried at temperatures of 5–20 °C for seven days, and ground for better manipulation and storage. Powdered samples (<1 mm) were stored in airtight bags in a refrigerator at −18 °C until use. 

### 3.2. Physicochemical Characterization of the Raw Material 

The main physicochemical properties of the raw material were obtained using international standard analytical procedures. Moisture and ash content were gravimetrically determined in triplicate by ISO 638 and 776:2021, respectively. Samples were placed in a convective oven at 105 °C for 48 h to determine the percentage of humidity. The samples were placed in a muffle furnace at 575 °C for 6 h to determine the ash content.

Total nitrogen was determined by mass spectrometry (FlashEA 1112 Elemental analyzer, Thermo, Waltham, MA, USA) and converted to protein using the universal factor 6.25. 

Mineral and heavy metals content (Ca, Mg, Na, Fe, Zn, Cu, Cd, and Pb) was determined by atomic absorption (SpectrAA-220 Fast Sequential, Varian, Palo Alto, CA, USA) and expressed as weight units.

Acid hydrolysis in two stages was carried out for the quantification of carbohydrates. In the first stage, samples were mixed with sulfuric acid (72%) for 60 min at 30 °C under agitation to break the polysaccharides. In the second stage, samples were placed in an autoclave for 60 min at 204 kPa with 4% sulfuric acid to obtain monosaccharides. The extracts were filtered through Gooch cresol (n3) and, for the determination of carbohydrates, were filtered again through a cellulose membrane of 0.45 µm. These samples were subsequently analyzed by HPLC, using an 1100 series Hewlett-Packard chromatograph with refractive index detector at 60 °C with a 300 × 7.8 mm Aminex HPX-87H column (Bio-Rad, Hercules, CA, USA) with a mobile phase of sulfuric acid (0.003 M) in a flow rate of 0.6 mL/min.

### 3.3. Extraction Technologies 

#### 3.3.1. Hot Pressurized Liquid Extraction with Microwave Heating (PHWE-MH)

**Figure 7:**
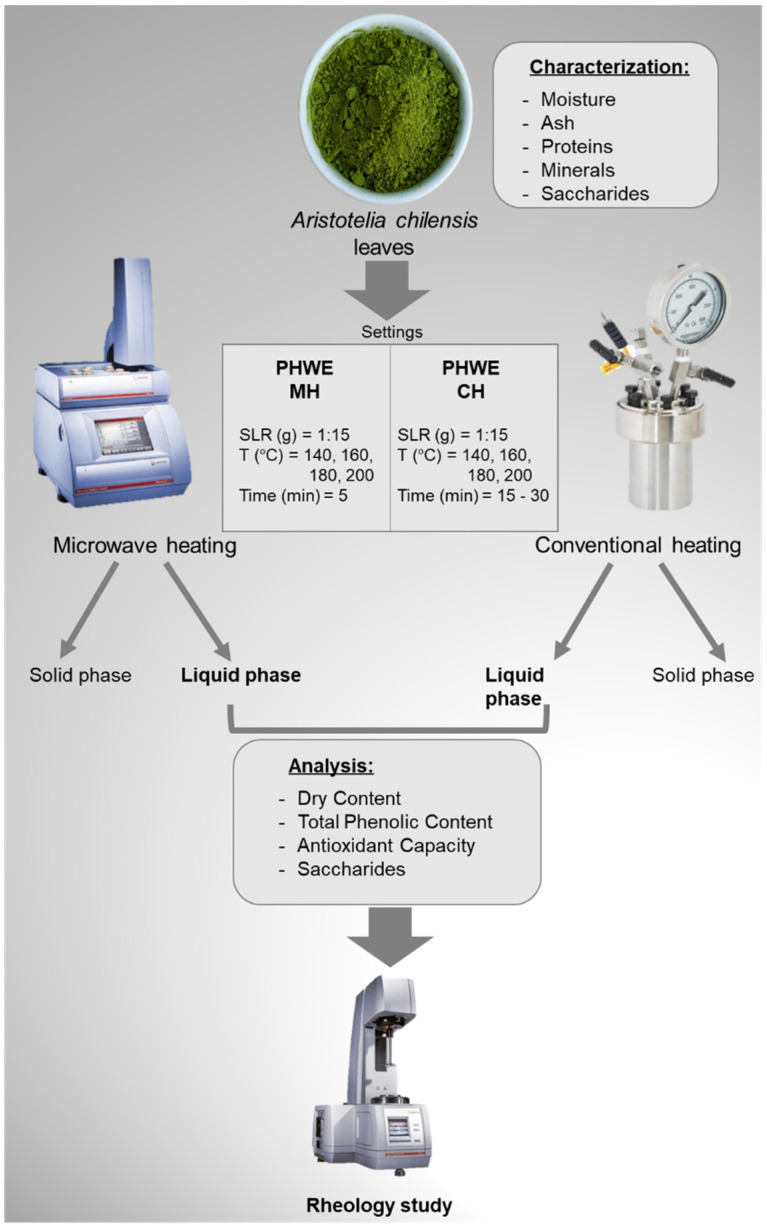
Scheme of the experimental and analytical approaches during the study of pressurized hot water extraction with microwave heating (PHWE-MH) and conventional heating (PHWE-CH) of bioactive compounds from *Aristotelia chilensis* leaves.

Extraction experiments were performed in triplicate in a Monowave 450 single-mode microwave oven (Anton Paar, GmbH, Graz, Austria) with an air compressor for cooling purposes, using distilled water as a solvent. One g of dry-weight leaves was mixed with 15 g of distilled water into a standard vial of 30 mL Pyrex vessel (G30) and taken to a temperature of 140, 160, 180, and 200 °C determined via IR detector for 5 min under constant stirring at 800 rpm; the power of the microwave oven was automatically set to achieve the time extraction heating curve and to maintain the temperature constant for the selected time. Extracts were subsequently vacuum filtered to separate liquid extracts from solid remains. Liquid samples were refrigerated for further analysis. A simplified scheme of the experimental and analytical approach is shown in Figure 7.

#### 3.3.2. Hot Pressurized Liquid Extraction with Conventional Heating (PHWE-CH)

Hot pressurized aqueous extraction under nonisothermal conditions was performed in duplicate in a continuously stirred pressure reactor of 100 mL volume, equipped with a controlled heater (Parr Instrument Company, Moline, IL, USA). An amount of 4 g of dry leaves was mixed at 150 rpm with 60 g of distilled water. When the target temperature was reached, the system was cooled by a continuous water circulation through a stainless steel coil. The obtained liquid phases were filtered, and liquors were cold-stored for further analysis.

Severity was estimated from the temperature evolution for both heating treatments following Equation (1) by Overend [26]:

(1) $$R_{0}=\int_{0}^{t}\exp\frac{T\left(t\right)-Tr}{W}\;dt$$

where R_0_: severity factor, *t*: reaction time (s); *T*: temperature (°C), *Tr*: reference temperature (°C), *W*: constant = 14.75 (activation energy of reaction).

### 3.4. Aqueous Extracts Analysis

The impact of the drying method before the analysis of the antioxidant features of the aqueous extracts obtained for both PHWE-MH and PHWE-CH was assessed. For this purpose, liquid extracts were freeze-dried (−55 °C, 0.97 mmHg, 5 days) and spray-dried (120 °C, feed speed: 20%, air flowrate: 40%, 2 h).

#### 3.4.1. Dry Weight Content

The dry weight of the liquors obtained in the range of temperatures tested was determined by a standard gravimetric method and expressed as mg/g extract. Aliquots of 1 g extract were placed in a convective oven for 24 h until reaching constant weight at 105 °C.

#### 3.4.2. Total Phenolic Content 

The Folin–Ciocalteu test [27] was used to determine the total phenolic content using gallic acid as a standard; all analyses were performed in triplicate. With this method, 0.25 mL of liquid sample was mixed with 1.875 mL of distilled water, 0.125 mL of Folin–Ciocalteu’s reagent (diluted 1:1 with distilled water), and 0.25 mL of Na_2_CO_3_ (10%) in a glass tube. The mixture was vortexed and incubated in darkness at room temperature for 60 min. Then, the absorbance was read at λ = 765 nm in a UV spectrometer (Evolution 201, Thermo Scientific, Waltham, MA, USA) in 3.5 mL optical glass cuvettes. Results were expressed as mg of gallic acid equivalents (GAE) per g of maqui leaves dry extract.

#### 3.4.3. Antiradical Capacity (TEAC Value)

The total antioxidant capacity of liquid extracts was determined against the ABTS (2,2′-azinobis-(3-ethyl-benzothiazoline-6-sulfonate)) radical, according to the Trolox Equivalent Antioxidant Capacity (TEAC) test by Re et al. [28]. The ABTS^+^ (2,20-azinobis (3-ethylbenzothiazoline-6-sulfonic acid)) was produced by the addition of 7 mM commercial ABTS and 2.45 mM of potassium persulfate diluted with phosphate-buffered saline (PBS) at pH 7.4 until the absorbance of the solution at 734 nm reached 0.7. In this method, 10 µL of the sample was mixed with 1 mL ABTS solution and incubated at 30 °C for 6 min. Then, absorbance was read in a UV spectrometer (Evolution 201, Thermo Scientific, Waltham, MA, USA) in a 1 mL quartz cuvette. All samples were analyzed in triplicate. Results were expressed as mg of Trolox equivalents (TEAC) per g of maqui leaves dry extract.

#### 3.4.4. Saccharides and Derivate Compounds Content

High-performance liquid chromatography (HPLC) was used to determine saccharidic and derivate groups fraction on a chromatograph (1100 series Hewlett-Packard) with a refractive index detector at 60 °C, using a column (300 × 7.8 mm, Aminex HPX-87H, Bio-Rad, Hercules, CA, USA) with a mobile phase of sulfuric acid (0.003 M) at 0.6 mL/min. One sample of the extracts was filtered through 0.45 µm membranes and used for direct HPLC determination of monosaccharides, furfural, hydroxymethylfurfural, and acetic acid. Another sample of the extracts was treated by a hydrolysis stage using sulfuric acid (4%) in an autoclave for 20 min at 121 °C (Mod 4001756, Selecta, Barcelona, Spain) and cooled in a stream of water. The analysis of this latter sample provided information on the monosaccharide content as a result of the increase in monosaccharide concentration caused by post hydrolysis. The liquid extract obtained from each sample was filtered through 0.45 μm syringe membranes and emplaced in vials. Subsequently, HPLC measurements were performed as previously described, where oligomers content was determined by the difference between monomers content in the autohydrolysis and the post hydrolysis content.

Saccharides content was calculated against calibrations prepared with solutions of known concentration of pure standards (glucose, xylose, rhamnose, arabinose, ribose, and formic and acetyl groups) (Supplementary Materials). The chromatographic peak areas from standards were compared with the sample peaks to calculate the saccharide and derivate groups concentration. All experiments were carried out at least in duplicate and expressed as a percentage of the dry weight of the extract.

### 3.5. Gelling Matrices

The liquid extracts obtained with both heating methods at the tested temperatures were used as solvents to prepare gelling matrices using a gelling agent of hybrid carrageenan (1.0%, in 0.1 M KCl). Hybrid carrageenan was extracted from *M. stellatus* following the optimized conditions previously described [25], and the biopolymer content was selected based on the results reported in previous studies in which conventional procedures were employed [29]. Solutions were made by dissolving the appropriate biopolymer and salt content in the extracted liquid phases at 80 °C under stirring for 30 min to ensure complete dissolution. Before rheological testing, systems were cold-stored for 24 h to ensure full gel maturation. Afterward, the viscoelastic profiles (G’, elastic modulus, and G’’, viscous modulus, vs. frequency) of formulated systems were monitored using a controlled-stress rheometer (MCR 302, Anton Paar, Graz, Austria). Gelling matrices were placed on the selected measuring geometry (i.e., sandblasted parallel plate, 25 mm diameter, 1 mm gap), sealed with light paraffin oil, and rested for 10 min at 25 °C. Then, stress sweep tests were carried out at the same temperature to define the linear viscoelastic region (< 35 Pa). Subsequently, frequency sweeps from 0.1 to 10 Hz at 15 Pa and above temperature were performed to determine the mechanical features of the gelled matrices.

### 3.6. Statistical Analysis

Experimental measurements were carried out at least in triplicate. Data were statistically analyzed using one-factor analysis of variance, ANOVA. Whenever the variance analysis exhibited differences among means, a post hoc Scheffe test was conducted to differentiate means (95% confidence, *p* < 0.05).

## 4. Conclusions

The use of environmentally friendly technologies for natural products extraction has different advantages, being an alternative for mitigating the limitations of conventional extractions and ensuring an alternative for biocompounds recovery. The selected green technologies use water as the only solvent, and due to operation under pressurized conditions, they reduce energy consumption and solvents and prevent toxic effluents formation, being strategies much more sustainable with the environment.

Both PHWE heating methods tested in this research are appropriate for recovering phenolic compounds with antiradical properties from maqui leaves. These extraction technologies allowed adding value to *A. chilensis* leaves from maqui industrial discards by recovering antioxidant soluble extracts. Microwave provides faster heating and is probably best suited for scaling up the extraction process to the commercial level. Although soluble extracts with slightly higher antioxidant capacity were obtained with conventional heating, these took much longer than microwave heating. The main advantage of the use of microwave-assisted extraction is the relative rapidity of bioactive compounds extraction in contrast with conventional pressurized liquid extraction. Therefore, the very short extraction period could limit the degradation of thermolabile bioactives, an effect that can occur in conventional pressurized hot water extraction working at relatively milder temperatures. Even so, the investment in green technologies and the implementation are still a challenge, and more cost–benefit studies are required to evaluate the performance of these green extraction methods and their technical and economic viability. 

Our results point out the relevance of the heating dynamics in PHWE, which has been overlooked in the field. The severity function or the average temperature may be better optimization variables than the nominal temperature in PHWE since processing times are short and heating dynamics are slow. The obtained liquid extracts were highly antioxidant, rich in polyphenolics, and suitable for enriching gel matrices. Nowadays, the latter is highly appreciated in the pharmaceutical, cosmetic, and food industries for developing new products that would improve our quality of life by using eco-friendly technologies with non-polluting solvents. 

## Figures and Tables

**Table 1:** Chemical characterization of *A. chilensis* dried leaves in db, except moisture.

	Moisture (%)	9.68 ± 0.12
	Ash (%)	6.43 ± 0.18
	Proteins (%)	15.15 ± 0.18 ^1^
**Minerals (mg/kg)**	Potassium	10,500 ± 380 ^1^
	Calcium	21,230 ± 300 ^1^
	Magnesium	1980 ± 10 ^1^
	Sodium	20.14 ± 0.99 ^1^
	Iron	237.9 ± 4.5 ^1^
	Zinc	12.33 ± 0.14 ^1^
	Copper	<7.0 ^1^
	Cadmium	<5.0 ^1^
	Lead	<10.0 ^1^
**Oligomeric carbohydrates and other groups (%)**	Total	37.64
	Glucose	15.68 ± 0.02
	Xylose	10.39 ± 0.00
	Rhamnose	2.31 ± 0.01
	Arabinose	2.44 ± 0.06
	Fucose	0.22 ± 0.04
	Ribose	0.79 ± 0.13
	Formic acid	3.94 ± 0.27
	Acetic acid	1.89 ± 0.20

^1^ Rivera-Tovar et al., 2021 [10].

**Table 2:** Severity values for different temperatures using microwave and conventional heating in PHWE.

Temperature (°C)	Microwave Heating	Conventional Heating
140	1.36 ± 0.06 ^a^	1.52 ± 0.08 ^a^
160	1.90 ± 0.02 ^b^	2.22 ± 0.05 ^a^
180	2.05 ± 0.01 ^b^	2.92 ± 0.06 ^a^
200	2.56 ± 0.03 ^b^	3.48 ± 0.09 ^a^

Data values in a row with different superscript letters (a,b) are significantly different at the *p* ≤ 0.05 level.

## Data Availability

Data are presented in the paper and available from the authors.

## Supplementary Materials

The Supplementary Materials are available online.

## Sample Availability

Samples from the compounds are available from the authors.

## References

[1] Misle E., Garrido E., Contardo H., González W. (2011). Maqui [*Aristotelia chilensis* (Mol.) Stuntz]-the Amazing Chilean Tree: A Review. J. Agric. Sci. Technol. B.

[2] Rodríguez K., Ah-Hen K.S., Vega-Gálvez A., Vásquez V., Quispe-Fuentes I., Rojas P., Lemus-Mondaca R. (2016). Changes in bioactive components and antioxidant capacity of maqui, *Aristotelia chilensis* [Mol] Stuntz, berries during drying. LWT-Food Sci. Technol..

[3] Zúñiga G.E., Tapia A., Arenas A., Contreras R.A., Zúñiga-Libano G. (2017). Phytochemistry and biological properties of *Aristotelia chilensis* a Chilean blackberry: A review. Phytochem. Rev..

[4] Insunza V., Aballay E., Macaya J. (2001). In vitro nematicial activity of aqueous plant extracts on chilean populations of *xiphinema americanum* sense lato. Nematropica.

[5] Essien S.O., Young B., Baroutian S. (2020). Recent advances in subcritical water and supercritical carbon dioxide extraction of bioactive compounds from plant materials. Trends Food Sci. Technol..

[6] Flórez N., Conde E., Domínguez H. (2014). Microwave assisted water extraction of plant compounds. J. Chem. Technol. Biotechnol..

[7] Samadian H., Maleki H., Allahyari Z., Jaymand M. (2020). Natural polymers-based light-induced hydrogels: Promising biomaterials for biomedical applications. Co-Ord. Chem. Rev..

[8] Anamica, Pande P.P. (2017). Polymer Hydrogels and Their Applications. Int. J. Mater. Sci..

[9] Rodríguez-Seoane P., Domínguez H., Torres M.D. (2020). Mechanical Characterization of Biopolymer-Based Hydrogels Enriched with Paulownia Extracts Recovered Using a Green Technique. Appl. Sci..

[10] Rivera-Tovar P.R., Torres M.D., Camilo C., Mariotti-Celis M.S., Domínguez H., Pérez-Correa J.R. (2021). Multi-response optimal hot pressurized liquid recovery of extractable polyphenols from leaves of maqui (*Aristotelia chilensis* [Mol.] Stuntz). Food Chem..

[11] Damascos M.A., Arribere M. (2009). Mineral content from *Aristotelia chilensis* (Molina) Stuntz leaves used in phytotherapy. Rev. Cuba. Plantas Med..

[12] Cespedes C., Jakupovic J., Silva M., Watson W. (1990). Indole alkaloids from *Aristotelia chilensis*. Phytochemistry.

[13] He K., Valcic S., Timmermann B.N., Montenegro G., Lkaloids I.N.A., Ristotelia F.A., Ol C.M. (1997). Indole Alkaloids from *Aristotelia chilensis* (Mol.) Stuntz. Int. J. Pharmacogn..

[14] Fredes C., Paz R. (2018). Maqui (*Aristotela chilensis* (Mol.) Stuntz). Fruit and Vegetable Phytochemicals: Chemistry and Human Health.

[15] Muñoz O., Christen P., Cretton S., Backhouse N., Torres V., Correa O., Costa E., Miranda H., Delporte C. (2011). Chemical study and anti-inflamatory, analgesic and antioxidant activities of the leaves of *Aristotelia chilensis* (Mol.) Stuntz, Elaeocarpaceae. J. Pharm. Pharmacol..

[16] Bacanli M., Bas A.A., Basaran N., Watson R., Preedy V., Zibadi S. (2018). A Triterpenoid Commonly Found in Human Diet: Ursolic Acid. Polyphenols: Prevention and Treatment of Human Disease.

[17] Vázquez-Espinosa M., Espada-Bellido E., de Peredo A.V.G., Ferreiro-González M., Carrera C., Palma M., Barroso C.G., Barbero G.F. (2018). Optimization of Microwave-Assisted Extraction for the Recovery of Bioactive Compounds from the Chilean Superfruit (*Aristotelia chilensis* (Mol.) Stuntz). Agronomy.

[18] Rubilar M., Jara C., Poo Y., Acevedo F., Gutiérrez C., Sineiro J., Shene C. (2011). Extracts of Maqui (*Aristotelia chilensis*) and Murta (*Ugni molinae* Turcz): Sources of Antioxidant Compounds and α-Glucosidase/α-Amylase Inhibitors. J. Agric. Food Chem..

[19] Devi R.A., Arumugam S., Thenmozhi K., Veena B. (2018). Phytochemical and in vitro antioxidant of an endemic medicinal plant species, *Elaeocarpus munronii* (WT.) Mast and *Elaeocarpus tuberculatus* Roxb. (Elaeocarpaceae). J. Pharmacogn. Phytochem..

[20] Kumar T.S., Shanmugam S., Palvannan T., Kumar V.B. (2008). Evaluation of Antioxidant Properties of *Elaeocarpus ganitrus* Roxb. Leaves. Iran. J. Pharm. Res..

[21] Huaman-Castilla N.L., Martínez-Cifuentes M., Camilo C., Pedreschi F., Mariotti-Celis M., Pérez-Correa J.R. (2019). The Impact of Temperature and Ethanol Concentration on the Global Recovery of Specific Polyphenols in an Integrated HPLE / RP Process on Carménère Pomace Extracts. Molecules.

[22] Mariotti-Celis M.S., Martínez-Cifuentes M., Huamán-Castilla N., Pedreschi F., Iglesias-Rebolledo N., Pérez-Correa J.R. (2018). Impact of an integrated process of hot pressurised liquid extraction–macroporous resin purification over the polyphenols, hydroxymethylfurfural and reducing sugars content of *Vitis vinifera* ‘ Carménère ’ pomace extracts. Int. J. Food Sci. Technol..

[23] Sanz V., Flórez-Fernández N., Domínguez H., Torres D. (2020). Clean technologies applied to the recovery of bioactive extracts from *Camellia sinensis* leaves agricultural wastes. Food Bioprod. Process..

[24] Liu H., Jiang N., Liu L., Sheng X., Shi A., Hu H., Yang Y., Wang Q. (2016). Extraction, Purification and Primary Characterization of Polysaccharides from Defatted Peanut (*Arachis hypogaea*) Cakes. Molecules.

[25] Nooeaid P., Chuysinuan P., Techasakul S. (2017). Alginate/gelatine hydrogels: Characterisation and application of antioxidant release. Green Mater..

[26] Overend R. (1987). Fractionation of Lignocellulosics by Steam-Aqueous Pretreatments (and Discussion). Philos. Trans. R. Soc. Lond..

[27] Singleton V., Rossi J.A. (1965). Colorimetry of Total Phenolics with Phosphomolybdic-Phosphotungstic Acid Reagents. Am. J. Enol. Vitic..

[28] Re R., Pellegrini N., Proteggente A., Pannala A., Yang M., Rice-Evans C. (1999). Antioxidant activity applying an improved ABTS radical. Free Radic. Biol. Med..

[29] Larotonda F.D.S., Torres M.D., Gonçalves M.P., Sereno A.M., Hilliou L. (2016). Hybrid carrageenan-based formulations for edible film preparation: Benchmarking with kappa carrageenan. J. Appl. Polym. Sci..

