# Supplementary material for: Pressurized Hot Water Extraction and Bio-Hydrogels Formulation with Aristotelia chilensis [Mol.] Stuntz Leaves
Source: Molecules. 2021 Oct 23;26(21):6402. doi: 10.3390/molecules26216402 (PMC8586920; doi:10.3390/molecules26216402)
Supplement: Supplementary file 1 [file molecules-26-06402-s001.zip › molecules-1377115-supplementary.pdf]

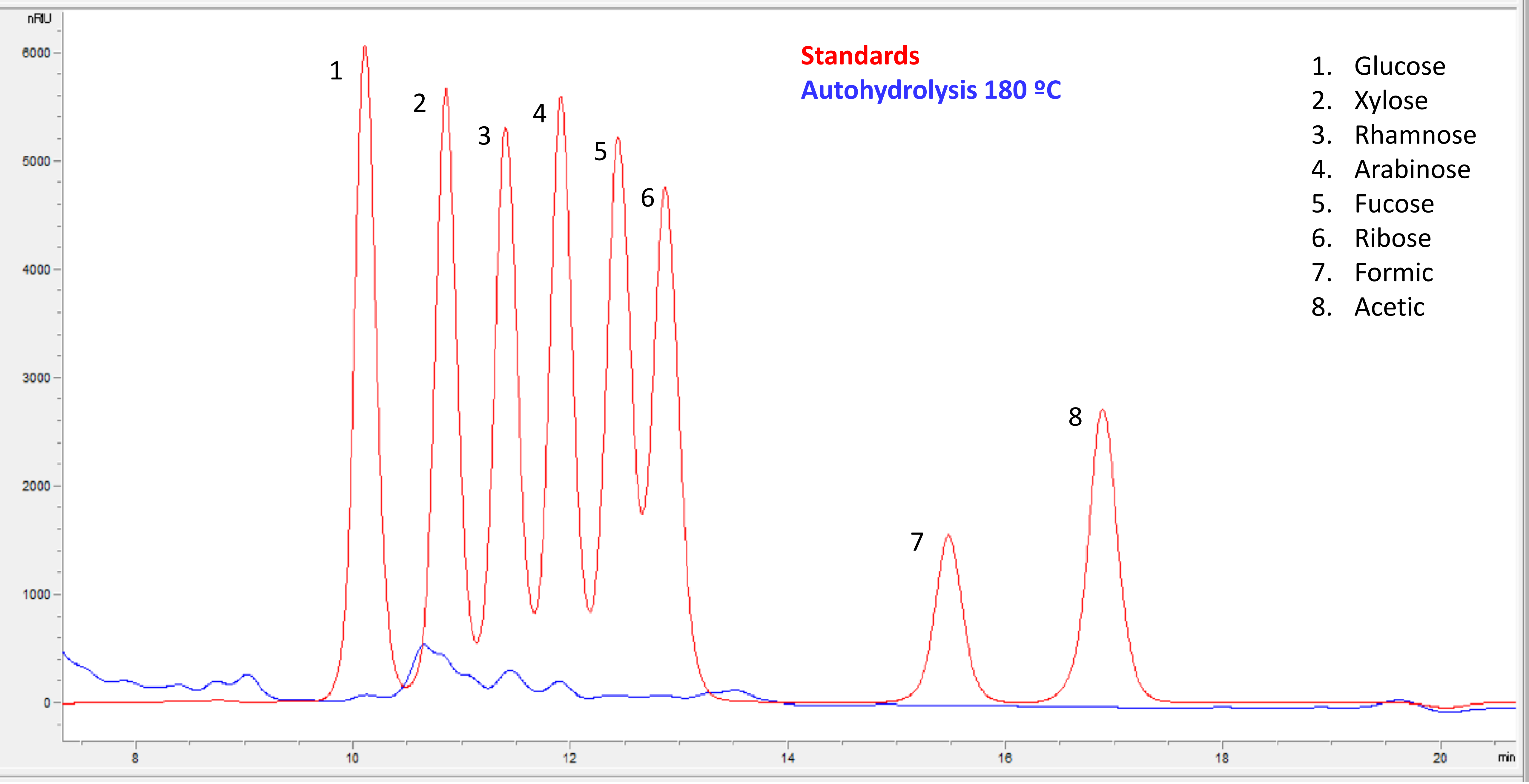

nRIU
6000
5000
4000
3000
2000
1000
0
8
10
12
14
16
18
20
min
1
2
3
4
5
6
7
8
Standards
Autohydrolysis 180 ºC
1. Glucose
2. Xylose
3. Rhamnose
4. Arabinose
5. Fucose
6. Ribose
7. Formic
8. Acetic

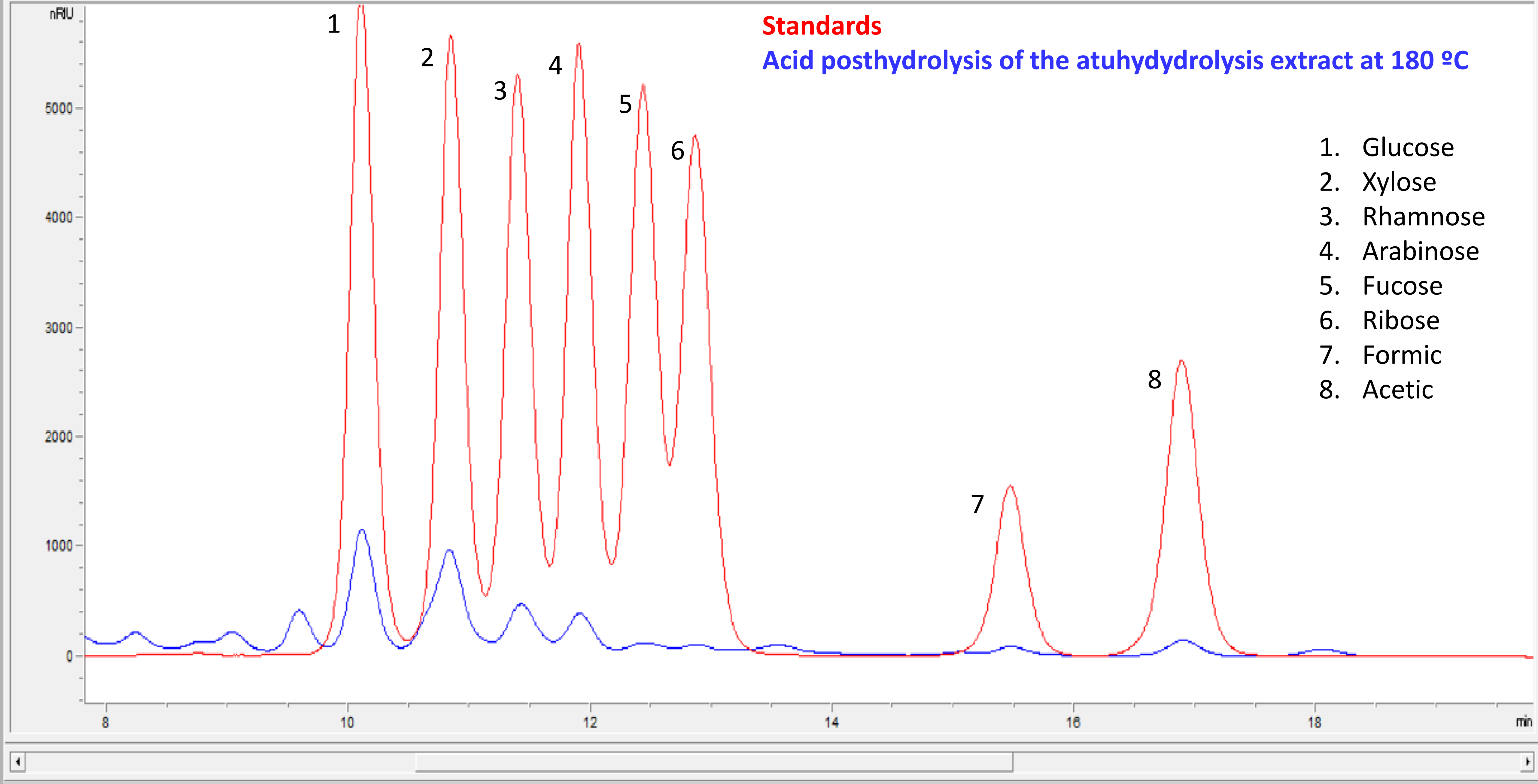

Standards
Acid posthydrolysis of the atuhydydrolysis extract at 180 ºC
1. Glucose
2. Xylose
3. Rhamnose
4. Arabinose
5. Fucose
6. Ribose
7. Formic
8. Acetic
nRIU
5000
4000
3000
2000
1000
0
8
10
12
14
16
18
min
1
2
3
4
5
6
7
8

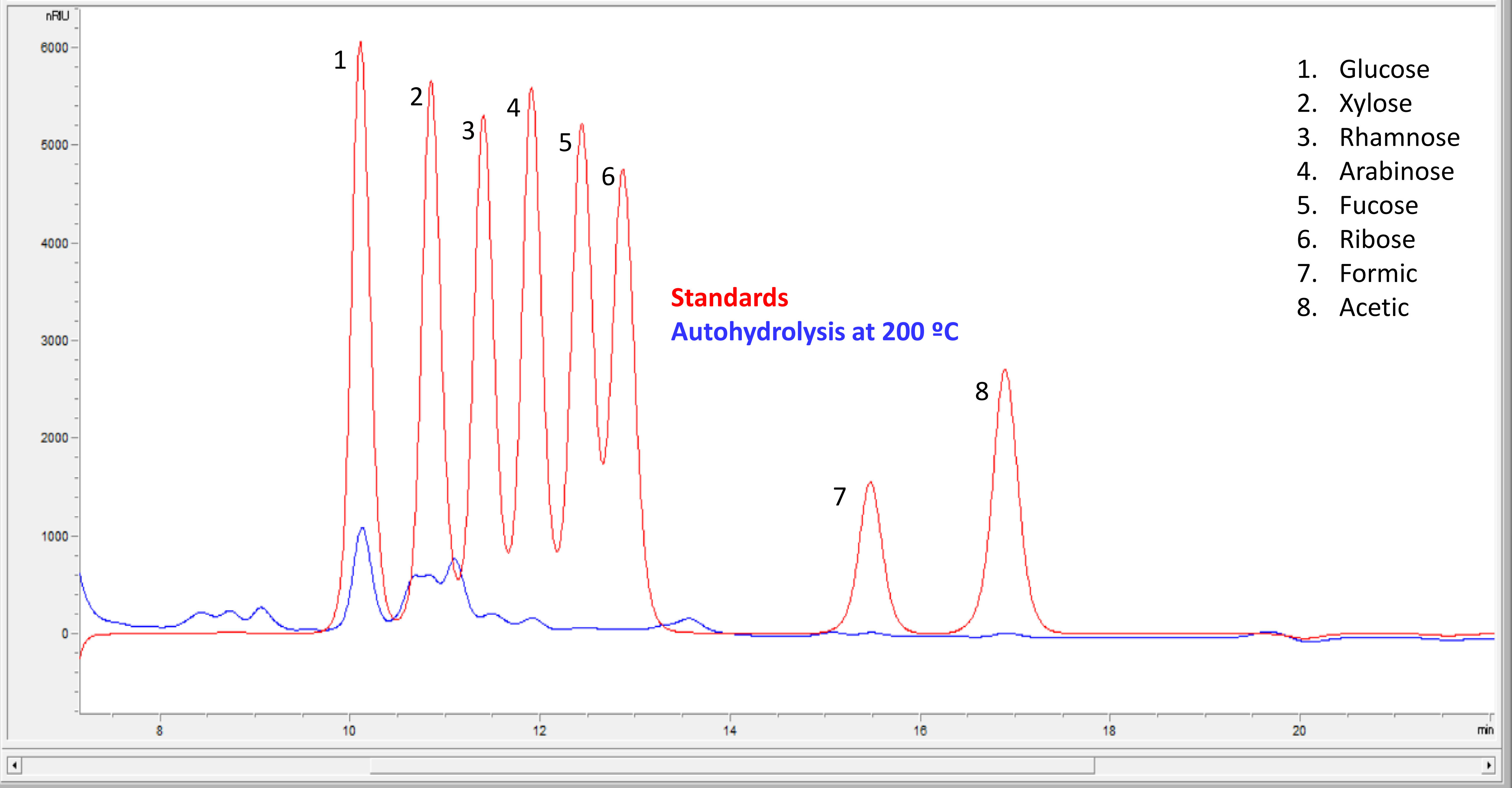
nRIU
6000
5000
4000
3000
2000
1000
0
8
10
12
14
16
18
20
min
1
2
3
4
5
6
7
8
1. Glucose
2. Xylose
3. Rhamnose
4. Arabinose
5. Fucose
6. Ribose
7. Formic
8. Acetic
Standards
Autohydrolysis at 200 ºC

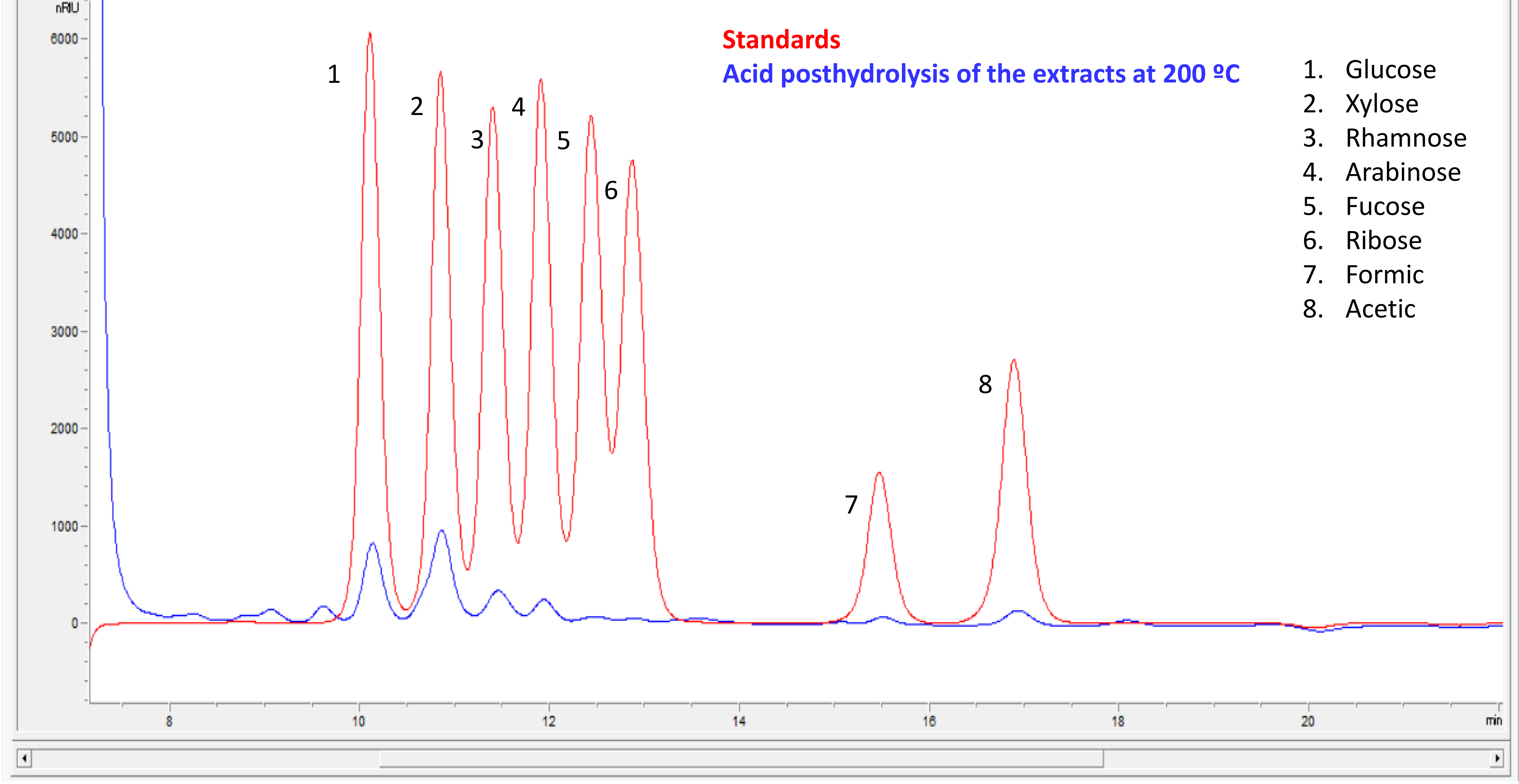

nRIU
6000
5000
4000
3000
2000
1000
0
8
10
12
14
16
18
20
min
1
2
3
4
5
6
7
8
Standards
Acid posthydrolysis of the extracts at 200 ºC
1. Glucose
2. Xylose
3. Rhamnose
4. Arabinose
5. Fucose
6. Ribose
7. Formic
8. Acetic

CALIBRATION

1. Glucose → $y=134452x + 13124$ $R^2 = 0.9908$
2. Xylose → $y=128898x + 12564$ $R^2 = 0.9913$
3. Rhamnose → $y=127223x + 11459$ $R^2 = 0.9896$
4. Arabinose → $y=133726x + 10861$ $R^2 = 0.9931$
5. Fucose → $y=138250x + 876.5$ $R^2 = 0.9954$
6. Ribose → $y=120921x + 12990$ $R^2 = 0.9951$
7. Formic → $y=19834x + 4221.5$ $R^2 = 0.9947$
8. Acetic → $y=37937x + 636.1$ $R^2 = 0.9964$
